# Alcohol-Metabolizing Enzymes, Liver Diseases and Cancer

**DOI:** 10.1055/a-2551-3320

**Published:** 2025-03-29

**Authors:** Tao Liu, FeiYu Zhang, Yue Feng, PanShiLi Han, YanHang Gao

**Affiliations:** 1 Department of Hepatology, Center of Infectious Diseases and Pathogen Biology, The First Hospital of Jilin University, Changchun, China; 2 China-Singapore Belt and Road Joint Laboratory on Liver Disease Research, Changchun, China; 3 Jilin Provincial Key Laboratory of Metabolic Liver Diseases, Jilin University, Changchun, China

**Keywords:** ADH, ALDH, CYP2E1, liver disease, liver cancer

## Abstract

Alcohol is generally believed to be metabolized in the liver by alcohol dehydrogenase (ADH), aldehyde dehydrogenase (ALDH), and to a much lesser extent cytochrome P450 2E1 (CYP2E1) and other enzymes. Recent studies suggest that gut also play important roles in the promotion of alcohol metabolism. ADH, ALDH, and CYP2E1 have several polymorphisms that markedly impact alcohol metabolism. These alcohol-metabolizing enzymes not only affect alcohol-associated liver disease (ALD), but may also modulate the pathogenesis of other liver diseases and cancer in the absence of alcohol consumption. In this review, we discuss alcohol metabolism and the roles of alcohol-metabolizing enzymes in the pathogenesis of ALD, metabolic dysfunction–associated steatotic liver disease, metabolic dysfunction and alcohol–associated liver disease, viral hepatitis, and liver cancer. We also discuss how alcohol-metabolizing enzymes may affect endogenous ethanol production, and how ethanol metabolism in the gut affects liver disease and cancer. Directions for future research on the roles of alcohol-metabolizing enzymes in liver disease and cancer are also elaborated.

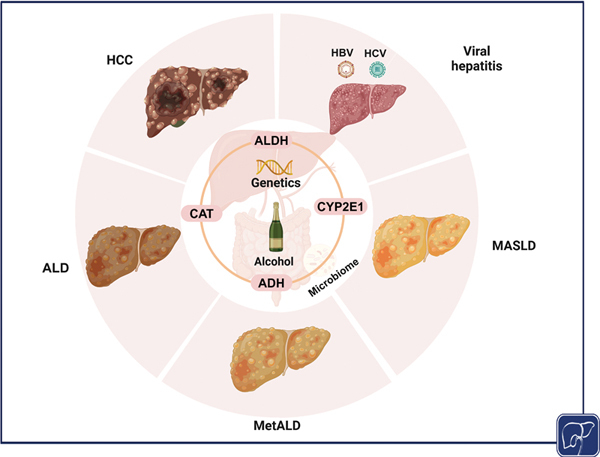

Alcohol consumption has severe impacts on human health. According to the World Health Organization (WHO), approximately 2.6 million people died due to harmful alcohol consumption, accounting for 4.7% of all deaths worldwide, in 2019. 1 The metabolism of alcohol involves the actions of various enzymes, mainly alcohol dehydrogenase (ADH), aldehyde dehydrogenase (ALDH), cytochrome P450 2E1 (CYP2E1), and catalase (CAT). 2 3 These enzymes play key roles in the oxidation of alcohol, affecting the rate of alcohol clearance in the body and the generation of its metabolites.

Alcohol consumption has been linked to more than 200 diseases, 1 with the liver being the primary target due to its central role in alcohol metabolism. Alcohol-associated liver disease (ALD) is widely recognized to be caused by long-term heavy drinking and is a major cause of chronic liver disease (CLD) worldwide. 4 Moreover, the active metabolites produced during alcohol metabolism, especially acetaldehyde, are significantly associated with carcinogenesis. 5 Against this backdrop, investigating the roles of alcohol-metabolizing enzymes in liver diseases and cancer is of particular importance. It not only aids the understanding of alcohol's effects on liver health, but also provides new perspectives for clinical prevention and treatment strategies. The present article focuses on the roles of these enzymes in the occurrence and progression of liver diseases and cancer, aiming to advance research and clinical interventions. We also discuss the potential effects of alcohol-metabolizing enzymes on endogenous ethanol and the contribution of gut ethanol metabolism to liver diseases.

## Alcohol Metabolism and the Enzymes Involved

### Alcohol Metabolism

**Fig. 1:**
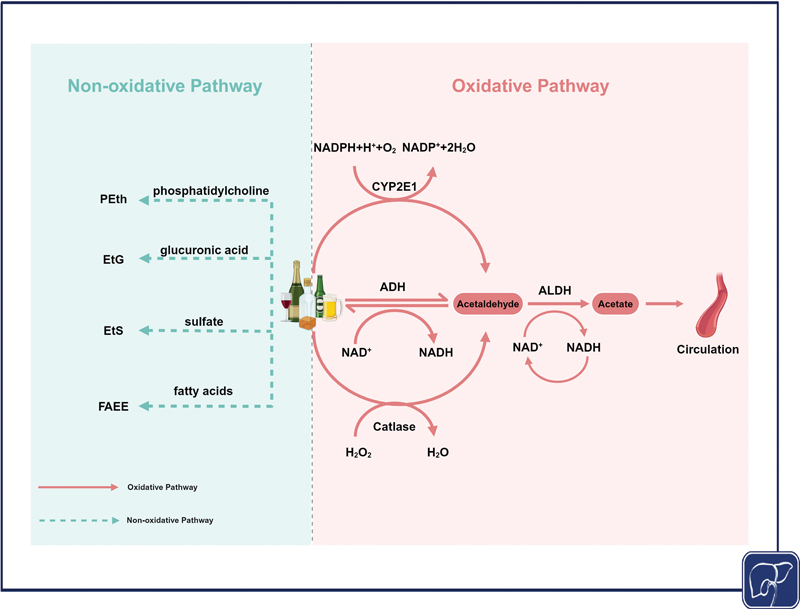
**Schematic of alcohol metabolism in the liver.** In the liver, alcohol is metabolized to acetaldehyde primarily via oxidative pathways by ADHs, CYP2E1, and CAT. Acetaldehyde is further metabolized by ALDH to acetate, which is subsequently released into the circulation and metabolized peripherally into carbon dioxide and water. Ethanol can also be metabolized through non-oxidative pathways, resulting in the formation of non-oxidative metabolites such as PEth, EtG, EtS, and FAEEs through interactions with phosphatidylcholine, glucuronic acid, sulfate, and fatty acids. ADH, alcohol dehydrogenase; ALDH, aldehyde dehydrogenase; CAT, catalase; CYP2E1, cytochrome P450 2E1; EtG, ethyl glucuronide; EtS, ethyl sulfate; FAEEs, fatty-acid ethyl esters; PEth, phosphatidylethanol. (Created in BioRender.)

After its ingestion, alcohol is absorbed into the bloodstream primarily through the stomach and the upper part of the duodenum, 6 with more than 90% of it being transported to the liver via the portal vein system. 7 8 The liver is the main organ for alcohol metabolism in the body; however, this traditional notion is challenged by recent studies showing that the gut–liver axis rather liver alone plays an important role in systemic acetaldehyde clearance. 9 Gut ethanol metabolism likely contributes significantly to alcohol-induced bowel disease. 10 In the liver, alcohol is metabolized through oxidative and non-oxidative pathways (see Fig. 1 for details). 7 11 12

Acetaldehyde, a highly reactive and toxic substance generated during alcohol metabolism, primarily causes DNA mutations, chromosomal damage, structural and functional impairments of organ, and the development of tumors by forming various adducts with DNA and proteins. 13 14 These protein adducts also upregulate CYP2E1 expression and enhance oxidative stress. 15 16 Non-oxidative pathway products also mediate ethanol-induced organ damage; for example, fatty acid ethyl esters induce endoplasmic reticulum stress (ERs), thereby promoting the occurrence of acute liver injury 17 and inducing mitochondrial dysfunction in hepatocytes and intestinal epithelial cells. 18 Non-oxidative metabolites are cleared more slowly from the body and can thus be used to assess recent alcohol consumption. 12 19

### Alcohol Dehydrogenase

ADH, a zinc-containing dimer enzyme located in the cytoplasm, is the most important enzyme in the metabolism of ethanol to acetaldehyde. 20 Based on its structural and kinetic characteristics, human ADH is classified into five types, with classes I, II, and IV being involved primarily in ethanol metabolism under physiological conditions. 21

Class I ADH, primarily expressed in the liver, stomach, colon, kidneys, and blood vessels, may be related closely to alcohol-induced flushing. 22 It is encoded by the genes *ADH1A* , *ADH1B* , and *ADH1C* , and genetic polymorphisms in *ADH1B* and *ADH1C* loci affect ethanol metabolism rate. Compared to ADH1B*1 carriers, ADH1B*3 carriers metabolize alcohol faster, which can reduce the risk of alcohol abuse. 23 Relative to carriers of other alleles, ADH1B*1 and ADH1C*2 carriers exhibit significantly slower ethanol metabolism, increasing the risk of alcohol use disorder (AUD) development. 24

Class II ADH is expressed mainly in the liver and has less affinity for ethanol than does class I ADH, with a correspondingly lesser role in ethanol metabolism. 25 Among ADH family members, only class IV ADH is not expressed in the liver, and it is most active in the presence of high ethanol concentrations. 26 Class IV ADH was recently detected in the esophagus and stomach and was found to be responsible primarily for first-pass ethanol clearance. 27 Approximately one-third of Asian individuals do not express it. 27

### Aldehyde Dehydrogenase

The human ALDH family has 19 members, among which mitochondrial ALDH2, ALDH1B1, and cytosolic ALDH1A1 are related closely to ethanol metabolism. 28 ALDH1B1 and ALDH1A1 have functions similar to those of ALDH2 but are expressed at much lower levels and have weaker affinities for acetaldehyde; thus, they typically play minor roles in acetaldehyde oxidation. 29 30 Low ALDH1A1 activity is associated with a mild alcohol flush reaction in European individuals but has little impact on drinking behavior. 26 31

ALDH2, the primary enzyme for metabolizing acetaldehyde, is expressed strongly in the liver and adipose tissue, and more weakly in the kidneys, lungs, stomach, and skin. 3 32 A genetic variant, ALDH2*2 (rs671), prevalent in East Asian populations, disrupts the ALDH2 tetramer and significantly reduces its ability to metabolize acetaldehyde. 33 34 Carriers experience discomfort (e.g., nausea and headache) after ethanol intake, which reduces their risk of AUD and acute alcohol-related disease development. Therapeutic approaches involving the inhibition of ALDH2's function have been explored for the treatment of AUD. 35 36

### Cytochrome P450 2E1

CYP2E1 oxidizes ethanol, 37 with CYP1A2 and CYP3A4 contributing to lesser extents. With low ethanol concentrations, CYP2E1 has low catalytic efficiency (Km = 10 mM) and accounts for about 10% of all ethanol metabolism; it may play a greater role in ethanol oxidation at higher alcohol concentrations. 38 Chronic alcohol consumption can induce the expression of CYP2E1, which may be associated with faster alcohol clearance in heavy drinkers. CYP2E1 is also involved in the oxidation of compounds such as benzene and acetone, and it exacerbates oxidative stress in hepatocytes by generating reactive oxygen species (ROS). 39 The expression of CYP2E1 is regulated by the ethanol concentration and can be modulated by cytokines such as interleukin-4 and miRNAs (e.g., hsa-miR-214-3p). 39 40 41 Due to the increased activity of CYP2E1 in heavy drinkers, the metabolism of certain drugs may be accelerated, increasing the risk of adverse reactions. 42

### Catalase

CAT is a tetrameric enzyme containing heme that metabolizes hydrogen peroxide (H _2_ O _2_ ) into oxygen and water and is distributed widely in various tissues. 43 It promotes ethanol oxidation by degrading H _2_ O _2_ in peroxisomes, but this pathway is not critical in hepatic ethanol metabolism. 11 CAT expression can be induced by long-term ethanol intake, especially under oxidative stress, with the ability of this enzyme to metabolize ethanol increasing with H _2_ O _2_ production. 16 44 CAT plays an important role in alcohol metabolism in the brain, and its metabolite acetaldehyde is considered to be a key factor in alcohol reinforcing effects, tolerance, and voluntary ethanol intake. These effects are likely related closely to the interaction of acetaldehyde with catecholamines to produce various condensation products. 38 45 46

## Alcohol-Metabolizing Enzymes and Liver Diseases

### Potential Effects of Gut Ethanol Metabolism on Liver Disease and Cancer

Ethanol in the intestine is primarily absorbed through the mucosa diffusion and metabolized by gut cells and microbiota. 11 The gut microbiota and enterocytes express alcohol-metabolizing enzymes, including ADH and ALDH, which co-metabolize ethanol into acetaldehyde and acetate. 47 ADH expression in the intestine exhibits a gradient, with higher levels in the proximal region and gradual reduction toward the distal intestine. Recent studies have challenged the view that the liver is the sole site of acetaldehyde metabolism, suggesting that gut–liver synergy is the primary mechanism for the clearance of acetaldehyde from the circulation. 9 Animal experiments have shown that dual ALDH2 knockout in the gut and liver synergistically reduces alcohol preference and intake relative to knockout in one of these organs. 9 Moreover, liver-specific ALDH2 inhibition alleviates heavy, but not moderate, drinking. 3 This finding provides a new perspective for the treatment of AUD and moderate drinking, which are common in patients with CLD.

Acetaldehyde, the primary metabolite of ethanol, is reintroduced into the intestinal lumen via bile. 9 Together with residual ethanol and acetate, it synergistically damages the intestinal barrier, disrupting tight junction integrity and increasing permeability. 48 Simultaneously, ethanol suppresses the expression of antimicrobial proteins, weakening microbial homeostasis. 49 50 These alterations promote the translocation of endotoxins (e.g., lipopolysaccharide [LPS]) into the portal circulation via the compromised barrier. 51 Following this translocation, microbial-associated molecular patterns and damage-associated molecular patterns from stressed and damaged cells can enter the liver through the portal vein, activating immune and parenchymal cells on the surfaces of toll-like and nucleotide-binding oligomerization domain-like receptors, triggering inflammatory responses, promoting hepatic fat accumulation, and accelerating liver fibrosis. 4 52 Notably, gut-derived ethanol (e.g., from high ethanol-producing *Klebsiella pneumoniae* strains) induces intestinal barrier damage and hepatic inflammation via mechanisms analogous to exogenous ethanol. 53 The bidirectional gut–liver axis interactions are experimentally validated in both ALD and metabolic dysfunction-associated steatotic liver disease (MASLD). 4 52 53

Approximately 30% of the acetaldehyde produced in the liver is secreted into the bile, where it is concentrated in the gallbladder, leading to further increase in acetaldehyde levels after alcohol consumption. 9 This process may partly explain the reported link between alcohol intake and an increased risk of gallbladder cancer (GBC). 54 However, a recent meta-analysis showed that the risk of GBC is slightly elevated in regular drinkers without a clear dose–response relationship. 55 This discrepancy may arise from bile dynamics: short-term alcohol consumption transiently raises acetaldehyde levels (later excreted), while chronic use prolongs gallbladder exposure, possibly increasing GBC risk.

Alcohol consumption slightly increases the risk of colorectal cancer (CRC). 54 56 Studies of ALDH2*2 and CRC have yielded inconsistent results. 56 57 58 Fu et al 9 demonstrated that acetaldehyde entering the gut via bile is metabolized primarily by intestinal ALDH2, with minimal reabsorption, while gut microbiota contributes secondarily. 9 Thus, the intestinal acetaldehyde exposure time and concentration in ALDH2*2 allele carriers may change with alcohol consumption. In addition to hosts' alcohol-metabolizing enzymes, the gut microbiota plays a role in alcohol metabolism. Although animal studies suggest chronic alcohol promotes rectal carcinogenesis through bacterial ethanol and acetaldehyde metabolism, 59 recent findings argue gut microbiota does not directly metabolize ethanol but responds to elevated circulating acetate. These interactions require further clarification. 60

### Alcohol-Metabolizing Enzymes and Associated Liver Diseases

#### Alcohol-Associated Liver Disease

ALD is the leading cause of CLD worldwide. Alcohol-related cirrhosis accounts for nearly 60% of cirrhosis cases in Europe, North America, and Latin America, and the number of deaths due to ALD has increased in developed countries in the past decade. 61 62 63 The promoter region (c-262, C > T) of the CAT-encoding gene not only affects CAT activity, but is also related closely to the susceptibility to and severity of alcohol dependence. However, evidence for an association between *CAT* gene polymorphism and ALD is currently lacking. 64 Epidemiological studies have shown that the frequency of the *CYP2E1 rs2031920 c2* allele is higher than that of the *c1* allele in patients with ALD, but its correlation with ALD remains uncertain. 65 Existing evidence suggests that the rate of alcohol metabolism by ADH is inversely proportional to the risk of AUD development in carriers. 2 20 This association may be related to the discomfort caused by acetaldehyde accumulation. 2 20 Yokoyama et al 66 found that the presence of slow-metabolizing ADH1B*1 increases the susceptibility to fatty liver in alcohol-dependent men in Japan. However, data on ADH mutations and ALD remain inconsistent, requiring further research. 67 68

The ALDH2*2 allele can reduce the risk of AUD and ALD development in carriers. A study conducted in Taiwan showed that this allele was significantly less frequent in patients with alcohol-associated cirrhosis and alcohol dependence than in controls (9 and 6% vs. 30%). 68 Tanaka et al 69 found that the frequency of the ALDH2*1/*1 genotype was significantly higher in Japanese patients with ALD than in controls (80.6% vs. 39.4%). Yokoyama et al 66 reported that the presence of the ALDH2*1/*2 genotype in alcohol-dependent Japanese men increased susceptibility to fatty liver. Targeted resequencing of genes in the ethanol metabolism pathway identified *ALDH1L2* (c.337, C > G) heterozygote was distributed significantly in patients with ALD, 70 but further research is needed to confirm its association with ALD.

**Fig. 2:**
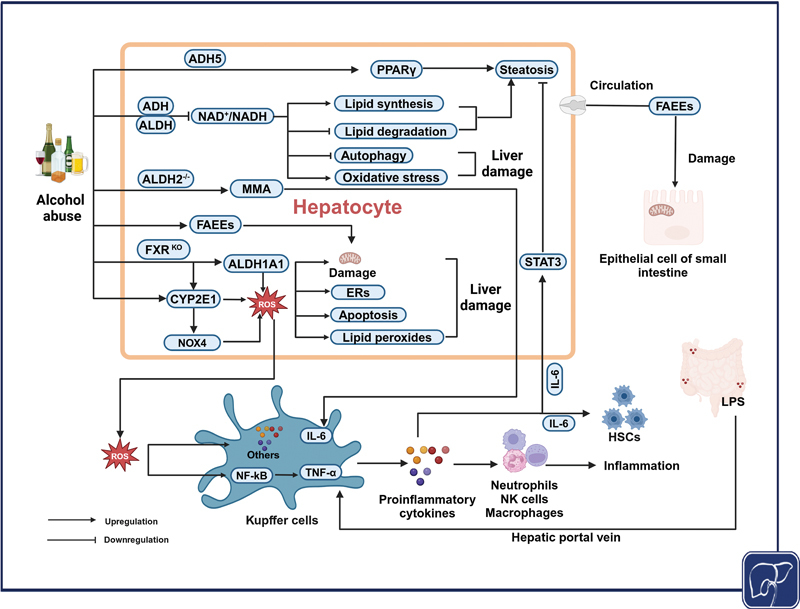
**The mechanisms by which alcohol-metabolizing enzymes are involved in alcohol-associated liver disease (ALD).** Alcohol abuse promotes the production of reactive oxygen species (ROS) through ALDH1A1 and CYP2E1. It induces endoplasmic reticulum stress (ERs), autophagy, lipid peroxidation, and mitochondrial damage, contributing to liver injury. FAEEs, as non-oxidative metabolites, also induce mitochondrial damage. In conjunction with acetaldehyde adducts such as MMA, they promote the release of pro-inflammatory cytokines, activating various immune cells and leading to inflammation. LPS translocation further stimulates the inflammatory response. Chronic alcohol consumption not only reduces the NAD +/NADH ratio, but also activates PPARγ through ADH5, increasing lipid synthesis and decreasing lipid catabolism, ultimately resulting in lipid accumulation. In ALDH2 knockout (ALDH2–/–) models, IL-6 levels are elevated, which reduces fat accumulation by inhibiting the expression of lipogenesis-related genes and stimulates the activation and proliferation of HSCs, promoting fibrosis. FAEE, fatty-acid ethyl esters; FXR, farnesoid X receptor; HSCs, hepatic stellate cells; IL-6, interleukin-6; LPS, lipopolysaccharide; MMA, malondialdehyde-acetaldehyde adduct; NAD +/NADH, oxidized/reduced nicotinamide adenine dinucleotide; NF-κB, nuclear factor-kappa B; NOX4, NADPH oxidase 4; PPARγ, peroxisome proliferator-activated receptor γ; TNF-α, tumor necrosis factor-α. (Created in BioRender.)

Chronic alcohol consumption upregulates CYP2E1, 40 promoting ROS production in hepatocytes and nicotinamide adenine dinucleotide phosphate oxidase 4 overexpression. This process activates nuclear factor-kappa B (NF-κB), increases the production of tumor necrosis factor-α (TNF-α), and enhances the sensitivity of Kupffer cells to LPS, driving ALD development. 71 ROS generated from alcohol metabolism in Kupffer cells activates inflammatory cytokines (e.g., TNF-α, interleukin [IL]-1β, and IL-6), triggering immune responses involving macrophages, neutrophils, and natural killer cells and thereby promoting liver damage. Additionally, ROS contributes to ALD progression by inducing mitochondrial damage, lipid peroxidation, ERs, and apoptosis. 18 72 73 With chronic alcohol consumption, the ADH/ALDH alcohol metabolism pathway becomes saturated, decreasing the oxidized/reduced nicotinamide adenine dinucleotide phosphate ratio, enhancing fatty acid synthesis (by increasing the expression of lipogenesis genes), reducing fatty acid oxidation, and deacetylating the transcription factor EB, which leads to reduced autophagy and increased oxidative stress. Furthermore, Haseba et al 74 found that ADH5 acts as an S-nitrosoglutathione reductase in mice fed alcohol for a long time, activating peroxisome proliferator-activated receptor γ (PPARγ) in the liver to promote lipid accumulation and facilitate ALD development ( Fig. 2 ).

ALDH2*2/*2 mice are more susceptible to alcohol-induced liver inflammation and fibrosis, but exhibit stronger resistance to alcohol-induced steatosis, which may be related to the activation of IL-6 expression in Kupffer cells by the malondialdehyde-acetaldehyde adduct. This activation, in turn, leads to the activation of signal transducer and activator of transcription 3 (STAT3) in hepatocytes, thereby inhibiting the expression of lipogenesis-related genes and promoting hepatic stellate cell activation and proliferation. 75 Guo et al 76 found that ALDH2 overexpression protected against cell apoptosis induced by long-term alcohol consumption. Morel et al 77 found that the farnesoid X receptor knockout mice had elevated oxidative stress, increased expression of ALDH1A1 and CYP2E1, and aggravated liver damage relative to controls. Their in-vitro experiments confirmed that ALDH1A1 activity is a key factor in alcohol-induced ROS generation. 77

Wang et al 78 found that CYP2E1-targeting RNAi delivered via lipid nanoparticles alleviated hepatic lipid accumulation, inflammation, and fibrosis in ALD mice, suggesting its therapeutic potential for ALD. After short-term exposure to large amounts of alcohol, the ADH metabolic capacity in the body becomes saturated. The targeting of ADH activation to accelerate alcohol metabolism is a promising therapeutic strategy for ALD. In recent years, various food proteins, traditional Chinese medicine components, and cell extracts have been found to enhance antioxidant, ADH, and ALDH2 activity, reducing liver steatosis, cell apoptosis, and alcohol-induced liver toxicity. 79 80 81 82 Given the important role of ALDH2 and the large population of mutation carriers, the targeting of ALDH2 is a promising therapeutic strategy for ALD. Selective small molecule ALDH2 activators have been reported to accelerate acetaldehyde clearance in mice and to alleviate liver steatosis and cell apoptosis. 83 84 However, the potential effects of ALDH2 activation on alcohol consumption limit the application of ALDH2 activators in ALD treatment. 85 Currently, no drug intended specifically for ALD treatment is available, and abstinence from alcohol remains the foundation of this treatment.

#### Metabolic Dysfunction–Associated Steatotic Liver Disease

MASLD (formerly known as non-alcoholic fatty liver disease [NAFLD]) has become the most common cause of CLD worldwide. 62 According to a recent meta-analysis, the global prevalence of MASLD has surpassed 30% and continues to increase. 86 Approximately 99% of patients diagnosed with NAFLD meet the criteria for MASLD. To avoid confusion, we use “MASLD” henceforth.

In addition to metabolizing alcohol, CYP2E1 is involved in ketone gluconeogenesis, fatty acid oxidation, and the detoxification of exogenous substances. 87 Studies of CYP2E1 mRNA and protein expression levels in the livers of patients with MASLD have yielded inconsistent results. 87 88 However, with the increasing prevalence of MASLD, 89 the changes in drug metabolism in this population are worth noting. Compared with those in healthy livers, the *CYP2E1* , *CAT* , *ADH* , and *ALDH* transcription levels are upregulated in the livers of patients with MASLD, 90 and these levels in patients with severe MASLD resemble those in patients with alcohol-associated hepatitis. Baker et al 91 observed significantly increased ADH1 and ADH4 protein levels in liver tissues from patients with metabolic dysfunction–associated steatohepatitis (MASH). However, Li et al 92 found that in MASH patients (stratified by liver fat content >5% or ≤5%), mRNA levels of alcohol-metabolizing enzymes remained unchanged compared to controls (except reduced ALDH4A1). Moreover, protein analysis revealed decreased CAT, ADH1A, ADH1B, and ADH4 and increased ALDH2 in both groups, while ALDH1A1 and ALDH1B1 reduction occurred only in the >5% fat group. 92 Moreover, in a population of mainly non-Hispanic white patients, compared with ADH1B*1 , ADH1B*2 was associated with a lower incidence of MASH and fibrosis. 93

In the absence of alcohol consumption, obese mice and obese patients with MASLD have significantly higher blood and exhaled ethanol concentrations than do controls, 94 95 which further rise with MASLD worsening and are reversible by antibiotic treatment. 96 97 Burger et al 98 recently found reduced ADH activity in the blood and livers of mice and patients with MASLD, potentially linking to TNF-α–c-Jun N-terminal kinase (JNK) pathway activation and subsequent ADH serine hyperphosphorylation. Treatment with TNF-α antibodies alleviates these changes in mice. 98 Current findings suggest complex associations between alcohol-metabolizing enzymes and MASLD. Further research is required to elucidate the relationships among alcohol consumption, alcohol-metabolizing enzymes, and MASLD.

#### Metabolic Dysfunction and Alcohol–Associated Liver Disease

Recently, a new nomenclature for fatty liver disease (SLD) introducing a new category of liver disease that involves alcohol and metabolic factors—metabolic dysfunction and alcohol–associated liver disease (MetALD)—was defined. 99 MetALD manifests as a spectrum from MASLD-dominant to ALD-dominant phenotypes. A recent US nationally representative data indicates a MetALD prevalence of 2.56%, with worse survival than MASLD. 100 101 Additionally, given the high underreporting of alcohol consumption in SLD populations, the true incidence of MetALD cases may exceed current estimates. 99 Although research remains limited, existing evidence underscores the critical role of alcohol-metabolizing enzymes in disease progression.

Numerous studies have revealed independent and complex relationships between metabolic risk factors included in the diagnostic criteria for MASLD and alcohol consumption. Alcohol intake is known to contribute to weight gain, 99 and 98% of heavy drinkers participating in one study had at least one cardiovascular metabolic risk factor, with more than 40% having four or more such factors. 102 Recent studies report that ADH1B*2 allele reduces ethanol-derived energy utilization, leading to lower weight gain in moderate drinkers compared to ADH1B*1 carriers. Notably, this effect is absent in non-drinkers, highlighting alcohol consumption as a prerequisite for the allele's metabolic impact. 93 A Scottish study linked BMI and alcohol consumption to liver disease, with the highest risk from BMI–heavy drinking (>120 g/week) interactions. 103 Separately, Japanese research found ALDH2*1/*2 genotype carriers with drinking habits exhibited elevated energy intake compared with ALDH2*1/*1 , potentially tied to acetaldehyde metabolism. 104

The relationship between alcohol consumption and type 2 diabetes (T2DM) is complex, with meta-analyses of Eastern and Western ethnic groups yielding conflicting results for moderate drinking. 105 106 This discrepancy may be related to genetic factors affecting alcohol-metabolizing enzymes. A recent meta-analysis of data from genome-wide association studies indicated that ALDH2*1/*1 is a susceptibility variant for T2DM in East Asian male populations. 107 Moreover, compared to ALDH2*2 carriers, ALDH2*1/*1 carriers increase alcohol intake, reduce fasting blood glucose clearance, and promote hepatic insulin resistance, elevating fasting glucose levels and T2DM's susceptibility. 108

Alcohol consumption correlates positively with hypertension, 109 which in turn is related closely to the *ADH1B* and *ALDH2* genotypes in Chinese individuals who consume alcohol, with the greatest hypertension risk seen in males carrying the ADH1B*1/*1 genotype. 110 Japanese men with the ADH1B *2/*2 genotype exhibited a stronger positive correlation between serum triglyceride levels and alcohol intake compared to ADH1B*1 allele carriers. 111 Additionally, compared to the ALDH2*1/*1 genotype, the ALDH2*2 allele is associated with lower high-density lipoprotein levels in alcohol-dependent and -independent manners. 112 113

#### Viral Hepatitis

The WHO estimates that 296 million and 58 million people worldwide were living with chronic hepatitis B (CHB) and C in 2019, respectively, with the highest disease burden being in Asia and Africa. 62 Alcohol use exacerbates liver injury in both infections: 26.5% of CHB patients report excessive alcohol consumption, rising to 35.6 and 41.8% in hepatitis B virus (HBV)-related cirrhosis and liver cancer patients, respectively, 114 while alcohol consumption synergistically exacerbates hepatitis C virus (HCV)-related liver damage. 115 116

Mechanistically, alcohol-induced CYP2E1 overexpression enhances HBV replication by upregulating hepatocyte nuclear factor-4α, the key transcription factor for the HBV core promoter, 117 and increases HCV-related mitochondrial ROS, reducing antioxidant capacity and depleting mitochondrial glutathione, which heightens oxidative damage and cell death. 118 However, both ethanol (via CYP2E1) and HBV can induce oxidative stress, complicating differentiation of their roles in liver damage. 119 120 Both the viruses interact with alcohol metabolism pathways: Patients with CHB carrying the ALDH2*2/*2 genotype are at greater risk of persistent HBV infection and high viral loads, and this genotype can predict the incidence of hepatocellular carcinoma (HCC) to some degree. 121 Whereas in HCV, the ADH/ALDH pathway may mediate the expression of interferon-stimulated genes via retinol (ROL) and retinoic acid (RA), thereby exerting antiviral effects. Alcohol may compete metabolically with ROL, weakening this antiviral function. 116 Despite these insights, the synergistic mechanisms between alcohol, metabolic enzymes, and viral hepatitis remain incompletely elucidated, necessitating further research.

## Alcohol-Metabolizing Enzymes and Liver Cancer

Recent data show that about 4.1% of cancers are alcohol related, causing significant health, economic, and social impacts. 122 Multiple studies have established dose–response relationships between alcohol consumption and the incidence of various cancers, including oral cancer, pharyngeal cancer, laryngeal cancer, CRC, HCC, and breast cancer. 5 123 124 The molecular mechanisms by which ethanol metabolism is linked to various cancer types, including liver cancer, have been recently reviewed. 125 Here, we focus on correlations between alcohol-metabolizing enzymes and tumors ( Table 1 ).

**Table 1:** Summary of alcohol-metabolizing enzymes and their associations with liver diseases and tumors

Enzyme name	Encoding gene	Primary function	Associated liver diseases/Tumors
ADH	*ADH1A*	Metabolizes ethanol, ROL, and other short-chain alcohols (e.g., methanol, ethylene glycol) 24	MASLD 91 92 ; HCC 146 147 148 149
	*ADH1B*	Metabolizes ethanol, fatty acids, acetone, epinephrine, glucose, ROL, tyrosine, tryptophan, ifosfamide, cyclophosphamide, abacavir, celecoxib, neurotransmitters serotonin, and norepinephrine 143	ALD, 66 67 68 MASLD 91 92 93 ; MetALD 93 110 111 ; HCC 141 146 147 149
	*ADH1C*	Metabolizes ethanol, ROL and other aliphatic alcohols, hydroxysteroids, and LPO products 150	MASLD 91 ; HCC 144 146 147 149
	*ADH4*	Oxidizes ethanol at higher concentrations; metabolizes ROL, tyrosine, fatty acid, drugs, and environmental toxicants 152	MASLD 91 92 ; HCC 149 151 152 153 155
	*ADH5*	Metabolizes formaldehyde and low affinity for ethanol; acts as a denitrating GSH reductase in ALD; exacerbates tetrachloride-induced liver fibrosis by increasing RA levels; protects MASLD by maintaining cellular GSH levels 74	ALD 74
	*ADH6*	Metabolizes ROL, ethanol, other aliphatic alcohols, LPO products, and hydroxysteroids 124	HCC 149
ALDH	*ALDH1A1*	Metabolizes ROL; oxidizes acetaldehyde, LPO-derived aldehydes, DOPAL; protects against ultraviolet-induced damage as lens and corneal crystallins; mediates a GABA synthesis pathway 36	ALD 77 ; MASLD 92 ; HCC 36 159 171 174
	*ALDH1B1*	Oxidizes acetaldehyde and LPO-derived aldehydes 36	MASLD 92 ; HCC 36 148 159 160
	*ALDH1L1*	Converts 10-fTHF to tetrahydrofolate 36	HCC 160
	*ALDH1L2*	Converts 10-fTHF to tetrahydrofolate 36	ALD 70
	*ALDH2*	Metabolizes acetaldehyde, DOPAL, and LPO-derived aldehydes; acts as a nitrate reductase 36	ALD 66 68 69 74 75 76 ; MASLD 92 ; MetALD 104 107 108 110 112 113 ; viral hepatitis 121 ; HCC 36 58 114 121 141 148 156 157 158 159 161
	*ALDH3A1*	Oxidizes aromatic, aliphatic aldehydes, and LPO-derived aldehydes; protects the cornea and lens against ultraviolet-induced oxidative stress 36	HCC 165 166
	*ALDH3B1*	Oxidizes LPO-derived aldehydes; involved in an alteration of dopamine metabolism 36	HCC 148
	*ALDH4A1*	Oxidizes glutamate γ-semialdehyde; oxidizes short- and medium-chain aliphatic LPO-derived aldehydes 36	MASLD 92
CYP2E1	*CYP2E1*	Metabolizes ethanol, catalyzes acetone, glycerol, different fatty acids, drugs, and environmental toxicants 39	ALD 65 71 77 ; MASLD 87 88 90 ; viral hepatitis 117 118 ; HCC 126 127 128 129 130 131 132 133 134 135 136 137 138 139
CAT	*CAT*	Dismutate of H _2_ O _2_ ; decomposes peroxynitrite; oxidizes nitric oxide to nitrogen dioxide; metabolizes reactive sulfide species 43	MASLD 90 92

Abbreviations: ALD, alcohol-associated liver disease; DOPAL, 3,4-dihydroxyphenylacetaldehyde; fTHF, 10-formyltetrahydrofolate; GABA, gamma-aminobutyric acid; GSH, glutathione; HCC, hepatocellular carcinoma; LPO, lipid peroxidation; MASLD, metabolic dysfunction-associated steatotic liver disease; MetALD, metabolic dysfunction and alcohol-associated liver disease; RA, retinoic acid; ROL, retinol.

### Cytochrome P450 2E1

Chronic alcohol intake accelerates CYP450-mediated (notably CYP2E1) chemically induced HCC in rodents, likely via enhanced activation of procarcinogenic toxins. 126 Studies on the relationship between the *CYP2E1 rs2031920* genetic polymorphism and HCC have yielded inconsistent results. Korean research identified higher prevalence of *CYP2E1 c1/c2* and *c2/c2* genotypes among drinkers, although no direct hepatocarcinogenic correlation emerged. 127 Contrastingly, another study has suggested the *CYP2E1 c1/c1* genotype in familial HCC susceptibility. 128 However, a Japanese study reported no association between *CYP2E1 (c1/c2)* genotype distribution and HCV-related HCC in patients versus controls. 129 The combined *ALDH2* ( *2/*2 ) *CYP2E1* ( *c1/c1* ) genotype was associated significantly with an increased risk of HCC. 129 Notably, Yu et al 130 demonstrated that the *CYP2E1 c1/c1* genotype amplifies the risk of HCC in smokers, further exacerbated by alcohol intake, highlighting the significant influence of gene–gene and gene–environment interactions. However, a recent meta-analysis revealed no association between the *CYP2E1 rs2031920* polymorphism and HCC susceptibility in East Asian populations. 131 Further large, well-designed studies are needed to clarify the inconsistent relationship between *CYP2E1* polymorphisms and HCC. In another *CYP2E1* polymorphic site, the *A1/A1* genotype of the variable number tandem repeat is associated with non-drinking and may reduce the risk of alcohol-related cancers in carriers. 132

The expression and activity of CYP2E1 are decreased in HCC tissue relative to those in adjacent normal liver tissue. 133 In real-time polymerase chain reaction analyses, Kinoshita and Miyata 134 and Ho et al 135 observed weak CYP2E1 mRNA expression in liver tissue from patients with HCC, which may be related closely to tumor invasiveness and poor prognosis. Additionally, patients with liver fibrosis and HCC showed more CYP2E1 activity than did controls. In rats, CYP2E1 activity significantly declined with liver fibrosis progression to HCC, yet higher activity correlated with more severe HCC, suggesting CYP2E1 hyperactivity is a risk factor for the progression of liver fibrosis to HCC. 136 Thus, CYP2E1 may play different roles in various stages of HCC development, and further in-depth research is needed.

CYP2E1 is involved in multiple signaling pathways. Abnormal activation of the Wnt/β-catenin signaling cascade is closely associated with the occurrence and development of HCC. 137 CYP2E1 overexpression triggers ROS accumulation, enhancing the interaction between disheveled segment polarity protein 2 (Dvl2) and Kelch-like protein 12 and thereby inducing the ubiquitination and degradation of Dvl2 and suppressing tumor growth via Wnt/β-catenin signaling. 133 Bile acids (BAs) are considered to be carcinogens that promote HCC. 138 Cholic acid (CA), one of the primary BAs synthesized in the liver, induces autophagy through the upregulation of protein kinase B (Akt) phosphorylation and downregulation of mammalian target of rapamycin (mTOR) phosphorylation and CYP2E1, promoting HCC cell growth and metastasis. 139 The upregulation of CYP2E1 in HCC cells inhibits this CA-induced autophagy and HCC cell growth via the Akt/mTOR pathway. 139 However, Ishteyaque et al 126 found that increased CYP2E1 levels stimulates the unfolded protein response and the upregulation of ERs-related proteins, suppresses the expression of B-cell lymphoma 2 (BCL2), and promotes HCC development via glucose-related protein 78/activating transcription factor 6 and CCAAT/enhancer-binding protein homologous protein signaling. These findings suggest that CYP2E1 plays a dynamic role in the pathogenesis of HCC and is a potential target for liver therapy.

### Alcohol Dehydrogenase

Epidemiological studies have revealed no independent correlation between HCC and *ADH* gene polymorphism. 58 140 141 142 143 However, the ADH1C*1/*1 genotype was found to increase the risk of HCC by 2.56 times in a population of 818 individuals with AUD (85 of whom had HCC). 144 *ADH* and *ALDH* interact synergistically. 145 A study conducted in Japan showed that ADH1B*2/*2 and ALDH2*1/*2 combined genotypes conferred a 2 to 4 times greater risk of HCC relative to other genotypes among light to moderate drinkers. 141 These findings indicate alcohol interacts with *ADH* / *ALDH* , urging medical professionals to closely monitor drinking habits, especially in East Asians.

Total and class-I ADH activities in cells are significantly elevated in primary and metastatic liver cancers, but activity in serum is elevated only in metastatic liver cancer. 146 147 In a proteomics analysis, Gao et al 148 classified patients with HBV-related HCC into three subgroups and found that ADH1A was associated significantly with differentiation and survival of this subgroup. Further validation in an independent cohort confirmed the association with survival, suggesting that ADH1A could serve as a robust prognostic marker. 148

Liu et al 149 found that high ADH1A, ADH1B, ADH1C, ADH4, and ADH6 expression levels were independent factors for improved HCC survival and prognosis. Gene enrichment analysis showed that low expression of these ADH genes correlated positively with pro-cancer signaling pathways. 149 150 Furthermore, compared with that in healthy liver tissue, the expression of ADH4 mRNA and protein in HCC tissue was found to be significantly reduced and to correlate positively with survival. 151 152 Tumor microenvironment (TME) immune cell infiltration and immune checkpoint expression affect the outcomes of immunotherapy for HCC. Li et al 153 recently found that ADH4 expression in HCC correlated negatively with the infiltration of immune cells (e.g., CD19+ B cells, CD68+ macrophages, and CD4+ T cells) and the expression of immune checkpoint proteins (e.g., cytotoxic T lymphocyte–associated antigen-4, programmed cell death protein 1, lymphocyte activation gene-3, and vascular endothelial growth factor B), suggesting that ADH4 is a potential target for immunotherapeutic intervention for HCC. Abnormal histone deacetylation is one of the mechanisms of immune evasion in HCC, 154 and the inhibitor trichostatin A (TSA) shows therapeutic potential. In a mouse model of c-Myc–induced HCC, ADH4 expression was regulated via the Akt–mTOR pathway, with the inhibition of adenosine triphosphate production and tumor growth, after TSA treatment. 155 Thus, ADH4 can serve as an independent prognostic marker and a potential therapeutic target in this context.

### Aldehyde Dehydrogenase

ALDH has been associated with the occurrence, development, and prognosis of HCC. 36 A Japanese study revealed that light to moderate drinkers with the ALDH2*1/*2 genotype had a significantly higher HCC risk compared with ALDH2*1/*1 carriers, 141 and this genotype is an independent risk factor for HCC development in patients with alcohol-associated liver cirrhosis. 156 A Taiwan study showed that heavy drinking and ALDH2*2 allele significantly increased HCC risk in HBV-related cirrhosis. 157 However, a large prospective study revealed no significant association between the *ALDH2* genotype and the HCC risk. 58 Moreover, in CHB patients, the *ALDH2* allele was not associated with HCC, but the ALDH2*2 allele combined with alcohol consumption was associated with reduced odds of HCC development. 158

Some studies have shown that ALDH1A1 expression is elevated and ALDH1B1 and ALDH2 expression is reduced in HCC tissues. 36 159 A recent proteomics and genomics analysis revealed that the protein expression levels of ALDH1B1, ALDH2, and ALDH3B1 are reduced, indicating the loss of liver-specific metabolic functions in patients with HBV-related HCC, and that this reduction may promote tumor progression by increasing the accumulation of exogenous compounds. 148 Stronger ALDH1B1 and ALDH1L1 expression has been associated with better clinical outcomes in patients with HBV-related HCC. 160 ALDH2 expression correlates negatively with HCC metastasis and invasion. 159 In addition, decreased ALDH2 levels and phosphorylation at S9 and S276 were associated with poor prognosis in patients with HCC. 161

Certain ALDH isoforms, such as ALDH1, ALDH3A1, and ALDH18A1, are overexpressed in tumor cells. 162 Such overexpression reduces ROS production in these cells, inhibits lipid peroxidation, and prevents the toxic aldehyde accumulation that triggers cell apoptosis. ALDHs also promote RA generation by immune cells (e.g., macrophages and dendritic cells), inducing, maintaining, and enhancing the function and stability of tumor-infiltrating regulatory T cells (Tregs), 163 while reducing ERs and immune cell death, 164 thereby weakening the immune response against tumors. The combined effects of these mechanisms provide survival advantages to tumor cells, promoting tumor progression, treatment resistance, and immune evasion. Significant ALDH3A1 upregulation in HCC and hepatocellular adenomas serves as a biomarker of Wnt/β-catenin signaling pathway activation, although no correlation has been found between ALDH3A1 expression and patient prognosis. 165 166

Our previous study showed that simple ALDH2 deficiency doesn't lead to liver disease progression, but increases HCC risk in patients with excessive alcohol consumption. 114 Prolonged exposure to alcohol leads to the release of oxidized mitochondrial DNA via extracellular vesicles in ALDH2-deficient mouse liver cells, and this DNA is transferred to nearby HCC cells and activates carcinogenic (JNK, STAT3, BCL2, and transcriptional coactivator with PDZ-binding motif) pathways, promoting the development of alcohol-related HCC. 114 AMP-activated protein kinase (AMPK) is associated with the occurrence and invasion of various tumors. Hou et al 159 reported that ALDH2 regulates acetaldehyde levels, alters the intracellular redox status, and activates AMPK and downstream signaling pathways, influencing the metastatic behavior of HCC. Tregs mediate immune tolerance in the TME and are related closely to poor prognosis. A recent multi-omics analysis found reduced ALDH2 levels and activity in HCC tissues and that ALDH2 overexpression inhibited Treg differentiation via the β-catenin/transforming growth factor-β1 signaling pathway, thereby suppressing HCC development. 161

Cancer stem cells (CSCs) have cancerous and hepatocyte-like characteristics and strong drug resistance, and are considered to be the root cause of HCC recurrence. 167 ALDH in various tumors has been identified as a CSC marker. High ALDH expression in CSCs contributes to chemotherapy and radiotherapy resistance. 168 CD133 +/ALDH(high) HCC cells are believed to have high tumorigenic potential, 169 and ALDH1 isoforms have been identified as stem cell markers associated with HCC tumorigenesis, metastasis, and chemotherapy resistance. 170 Previous study showed that ALDH1A1 overexpression in HCC-derived CSCs promotes sorafenib resistance, 171 while reducing the expression of several stem cell markers, including ALDH, could reverse the sorafenib-resistant HCC cell lines. 172 Zhang et al 173 recently reported that disulfiram/Cu reduced ALDH activity in HCC cells, thereby decreasing cell stemness and sorafenib resistance, which enhanced the cytotoxic effect of sorafenib. However, Suzuki et al 174 found that strong ALDH1A1 expression was associated with better tumor differentiation and had little correlation with stem cell characteristics in HCC cells. Tanaka et al 175 reached similar conclusions. Given the limited research on the relationship between ALDH and liver CSCs and the inconsistency of findings, further studies are needed to clarify this relationship and the potential application of ALDH inhibitors in liver cancer treatment.

## Potential Effects of Alcohol-Metabolizing Enzymes on Endogenous Ethanol Production: Controversial Data

Endogenous ethanol is generally believed to be produced by gut microbiota and to interact with hosts' health in complex ways. 97 In healthy individuals, common gut bacteria produce certain amounts of ethanol; those with metabolic diseases (e.g., T2DM, MASLD) or auto-brewery syndrome show significantly elevated endogenous ethanol. 53 176 177 However, due to the liver's first-pass metabolism, the amount of endogenous ethanol produced is difficult to infer from the peripheral circulation. Blood ethanol levels are elevated in MASLD patients. 96 178 The chronic overproduction of endogenous ethanol and its metabolites not only induces hepatic metabolic dysregulation and the development of liver disease, but may also exacerbate neurodegenerative diseases through brain inflammation. 97

Mechanisms of endogenous ethanol production in MASLD remain controversial. Alcohol-producing *Klebsiella pneumoniae* (found in up to 60% of MASLD patients) can induce MASLD in mice. 53 In contrast, Engstler et al 178 suggested that the elevated plasma ethanol in children with MASLD relative to healthy controls are associated closely with obesity indices and insulin resistance, not gut bacterial overgrowth. Furthermore, genetically obese (ob/ob) mice show similar portal vein and intestinal ethanol levels to lean mice, higher systemic ethanol, and reduced hepatic ADH and CYP2E1 activity. 178 Based on these results, the insulin-dependent impairment of ADH activity in the liver, rather than endogenous ethanol production, elevates blood ethanol in MASLD, a view also supported by Burger et al. 98

Studies report inconsistent data on alcohol-metabolizing enzyme expression (ADH, CYP2E1, CAT) in MASLD, 90 91 92 178 highlighting a complex relationship between alcohol-metabolizing enzymes and MASLD. Overall, the elevation of ethanol levels in the circulation under pathological conditions may stem not only from endogenous production but also liver metabolic dysfunction and insulin dysregulation.

## Conclusion

Although a substantial amount of research has focused on the roles of alcohol-metabolizing enzymes in liver diseases and tumors, many questions remain unanswered. Due to genetic factors, the expression and function of these enzymes differ among individuals. The impacts of these differences on alcohol's effects on the liver and tumorigenesis are not yet fully understood. Further research can help clarify these enzymes' functional variations in different populations and their interactions with alcohol, genes, and environmental factors. In addition, the regulatory mechanisms of alcohol-metabolizing enzymes, their relationships to oxidative stress and immune responses, and their potential as therapeutic targets for liver diseases and tumors are important areas for future research.
